# Unexpected and Rare Sites of Metastasis in Oncologic Patients

**DOI:** 10.3390/jcm12206447

**Published:** 2023-10-10

**Authors:** Walid Shalata, Ashraf Abu Jama, Amjad Abu Salman, Mitchell Golosky, Adam Solomon, Omar Abu Saleh, Regina Michlin, Sondos Shalata, Abed Agbarya, Alexander Yakobson

**Affiliations:** 1 The Legacy Heritage Cancer Center and Dr. Larry Norton Institute, Soroka Medical Center, Beer Sheva 84105, Israel; ashrafag@clalit.org.il (A.A.J.);; 2 Faculty of Health Sciences, Ben-Gurion University of the Negev, Beer Sheva 84105, Israeladamso@post.bgu.ac.il (A.S.); 3 Cardiology Division, Soroka Medical Center, Beer Sheva 84105, Israel; 4 Medical School for International Health and Sciences, Ben-Gurion University, Beer-Sheva 84105, Israel; 5 Department of Dermatology and Venereology, The Emek Medical Centre, Afula 18341, Israel; 6 Nutrition Unit, Galilee Medical Center, Nahariya 22000, Israel; sondoss2@gmc.gov.il; 7 Department of Oncology, Bnai Zion Medical Center, Haifa 31048, Israel

**Keywords:** colon cancer, rare metastasis, oncologic patients, male breast cancer, second primary, pancreatic cancer, melanoma

## Abstract

Case studies of rare oncologic metastases are an important source of clinical data for health care professionals and researchers. While infrequent, the knowledge base and clinical recommendations derived from such cases aid in advancements in the field. As such, we aim to add five cases to the growing body of literature. The first two male patients, aged 69 and 73, were diagnosed with colon adenocarcinoma, suspected to be a second primary prostate carcinoma, following positron emission tomography-computer tomography (PET-CT). This suspicion was ruled out by prostatectomy and histopathological investigations, which instead found adenocarcinoma of colonic origin. The next two male patients, ages 63 and 68, were diagnosed, respectively, with metastatic pancreatic adenocarcinoma with cardiac metastases and metastatic melanoma with distant metastases to the pancreas. The final patient was a 73-year-old male diagnosed with metastatic breast cancer after a radiological investigation of suspected renal cell carcinoma.

## 1. Introduction

Cancers affecting the colon, rectum, and anus are commonly grouped under the term “colorectal cancer” in medical literature to ensure consistency. From a global perspective, the prevalence of colorectal cancer ranks third, after lung and breast cancers, in terms of incidence. With respect to fatalities; however, colorectal cancer takes a more significant toll, second only to lung cancer as the leading contributor to global cancer-related deaths [1]. Men diagnosed with colorectal cancer face a markedly increased probability of subsequently developing a second primary prostate cancer, as indicated by a hazard ratio (HR) of 2.30 with a confidence interval (CI) of 95% ranging from 2.18 to 2.43 (*p* < 0.001). Intriguingly, this risk surpasses that of developing a second primary colorectal cancer. According to some research findings, this phenomenon is particularly evident among male patients below the age of 65 [2,3]. Men who experienced the emergence of second primary prostate tumors following an initial diagnosis of colorectal cancer were more likely to die from prostate cancer [2]. Approximately half of colorectal cancer patients will develop metastases [4]. Collectively, these distant metastases are recognized as a significant challenge to the success of treatment, consequently diminishing the overall prognosis for patients [4]. The liver is known to be the predominant location for metastatic spread, exhibiting a frequency ranging from 30% to 70%, followed by the lung with a frequency of 20% to 40%, and the bone with a lower incidence ranging from 5% to 10% [4]. Presently, instances of the prostate being identified as a verified site for colorectal metastases remain uncommon. Instead, it is frequently interpreted as a distinct second primary cancer, largely attributed to the substantial annual occurrence of prostate adenocarcinoma (approximately 180,000 cases per year) [5,6,7,8]. Nevertheless, aside from occurrences of secondary primary prostate adenocarcinoma, inadequate attention is given to the instances of prostate involvement in the context of metastatic colorectal cancer [9,10,11,12,13].

Pancreatic cancer, while less frequent than colorectal cancer, is considerably more pernicious and lethal on a case-by-case basis. In fact, according to recent trends, the incidence and mortality rates of pancreatic cancer are predicted to increase in the following decades. Specifically, pancreatic cancer deaths surpassed those of breast cancer in 2016 and are expected to become the second-highest cause of cancer death by the year 2030 [14,15]. Adenocarcinomas of the head of the pancreas are particularly aggressive, as cases often only present due to the symptoms of common biliary duct obstruction [16]. By this time, the cancer is exceedingly difficult to treat, both medically and surgically, with as much as 50% of patients also presenting with liver metastases [17]. Lung metastases are also common, either directly or via liver metastasis [18]. Interestingly, hyperlipidemia is a risk factor for metastasis to the lung (OR 2.30, 95% CI 1.02 − 5.25; *p* = 0.0445) [18].

The amorphous nature of pancreatic tumors, growing around major vessels, generally makes resection impossible without prior radiation or chemotherapy [18]. Only 10–15% of pancreatic cancers are resectable at the time of diagnosis [19]. Unfortunately, the 5-year survival rate for advanced disease has seen practically no change, with treatment only improving results by 0–10% [20]. Whereas 5-year survival in unresected patients remains under 10%, resected patients have a 5-year survival of about 20% [19]. For advanced disease, standard chemotherapies are chemotoxic drug combinations such as leucovorin, 5-fluorouracil (5-FU), oxaliplatin, and irinotecan (FOLFIRINOX) [21]. However, this treatment regimen only increases survival by 2% in metastatic disease [22]. Leucovorin, while used to reduce the toxicity of dihydrofolate reductase by supplementing growing cells with folic acid, was also shown to increase the efficacy of 5-FU in pancreatic adenocarcinoma and metastatic colorectal cancer [23,24]. Metastatic melanoma is also a significantly aggressive disease; however, expanding treatment options have steadily improved survivability [25]. Immune checkpoint inhibitors, such as Ipilimumab, an antibody against CTLA-4, and Nivolumab, an antibody against PD-1, have been used to unleash the ability of T cells to target neoantigen-presenting tumor cells, increasing the 5-year survival rate to 44% in Nivolumab, 26% in Ipilimumab, and 52% in the combination therapy in recent clinical trials [26]. Positive prognostic factors include low to normal LDH, low tumor burden, and the presence of a BRAF-activating mutation [25]. Additional research is needed on the viability of tyrosine kinase inhibitors, which limit tumor cell survival and proliferation, although combination therapy has shown the greatest promise due to acquired resistance to molecular targets and immune checkpoint inhibitors [27,28]. In the phase 3 COLUMBUS clinical trial for combination tyrosine kinase inhibitors, an overall survival rate of 35% was achieved in BRAFV600 mutant metastatic melanomas [29]. Additionally, BRAF and MEK inhibitors have shown long-term benefits because of their direct immune-modulating effects, including immune surveillance [30]. However, the adverse effect profiles of these drugs are higher than those of immune checkpoint inhibitors [31].

Metastatic melanoma has shown responsiveness to immunotherapy, as increased immune surveillance allows for a more robust inflammatory potential in the tumor microenvironment. This phenomenon is explained by inhibition of the innate immune-suppressive qualities of malignant cells, as compared to the limited reactivity of pancreatic adenocarcinoma, a so-called “cold” tumor [32]. The immune checkpoint inhibitors have increased survival and decreased further metastases to the brain [31]. For this reason, as treatment options potentially progress for metastatic melanoma and other reactive cancers, more work needs to be conducted to develop therapies beyond resection for pancreatic cancer. Therefore, current research is dedicated to neoadjuvant immunotherapy techniques without success [33]. The disease can spread to virtually any organ, most commonly presenting in the skin, lymph nodes, lung, brain, liver, and bones [34].

Breast cancer is extremely uncommon in male patients, accounting for around 1% of all cancers in men and less than 1% of all breast cancer cases [35]. Owing to the rarity of this condition, there has been a paucity of clinical trials dedicated to male breast cancer. As a result, the body of data and medical literature on this subject remains limited, primarily comprising a scant number of available case reports. The scarcity of comprehensive, relevant information has consequently led to the practice of basing treatment modalities for male patients on extrapolated insights drawn from the management and outcomes of female breast cancer cases [36,37]. Derived from crucial findings in a recent significant study encompassing a cohort of over 770 instances of male breast carcinoma, it was observed that a notable 98% of cases were categorized as primary cancers. Among the subset of cases that were evaluated as potential metastases arising from a distinct site of origin, a substantial majority of 58% were identified as originating from cutaneous melanomas [36]. In the context of the studied male breast cancer cohort, it was discovered that primary cases exhibited metastatic behavior in 26% of the patients under investigation. Notably, subsequent disease recurrence occurred in 56% of cases within the skeletal framework, 51% within the pulmonary system, and 17% within the hepatic domain [38]. Consequently, male patients diagnosed with breast cancer are overwhelmingly characterized by primary tumor status [39]. Furthermore, the occurrence of metastases affecting the male breast is notably five to six times less frequent compared to females [40]. An illustrative case study documented the instance of an 80-year-old male patient diagnosed with metastatic adenocarcinoma originating from the prostate, which subsequently extended to the right breast. Notably, this presentation initially posed diagnostic confusion due to its resemblance to gynecomastia. [41]. Findings from a study conducted in 2007 revealed that up to 12% of primary male breast cancers exhibit a notable occurrence of a secondary primary breast tumor. This underscores the importance of intensified screening measures for males diagnosed with breast cancer. Notably, the prevalent secondary primary malignancies included subsequent breast cancer (Standardized Incidence Ratio [SIR] = 52.12, 95% Confidence Interval [CI] = 31.83 − 80.49), cutaneous melanoma (SIR = 2.98, 95% CI = 1.63 − 5.00), and stomach cancer (SIR = 2.11, 95% CI = 1.01 − 3.88) [2,3,42].

An analysis of existing literature revealed a mere five documented cases involving patients with colon cancer that had progressed to prostatic metastasis. Notably, the earliest and most recent instances of such occurrences were diagnosed and reported in 1993 and 2013, respectively [9,10,11,12,13].

## 2. Rare and Exclusive Cases Description

### 2.1. A 69 Year Old Male with Colon Cancer Metastasized to the Prostate Gland

The patient is a 69-year-old male with a 35 pack years (PY) smoking history and hyperlipidemia who presented with abdominal pain and melena. He has no family history of cancer. In October 2015, he was diagnosed with stage 4 colon adenocarcinoma (T3 N2 M1) with peritoneal metastasis. In November 2015, the patient underwent a left hemicolectomy and further histological investigation of a subdiaphragmatic nodule. He began chemotherapy under the FOLFOX protocol with the inclusion of bevacizumab (Avastin). Specifically, the intravenous (IV) regimen incorporated bevacizumab 5 mg/kg, oxaliplatin 85 mg/m^2^, leucovorin 400 mg/m^2^, and 5-FU 400 mg/m^2^ on day 1, followed by a continuous IV infusion of 5-FU 2400 mg/m^2^ over 46 h every two weeks as adjuvant therapy for six cycles. One month after completing this treatment, the patient underwent exploratory laparotomy, in which no malignancies were found.

**Figure 1:**
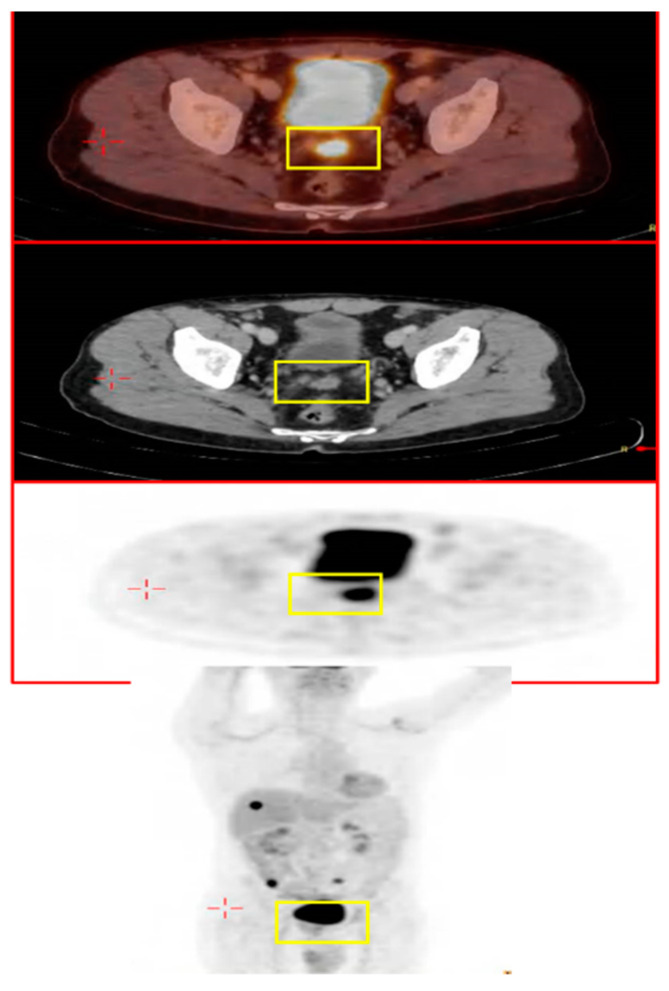
Images of the patient’s PET-CT showing the hyper-metabolic uptake in the area of the prostate (yellow squares).

The patient was examined two years later (April 2017). PET-CT showed areas of hypermetabolic uptake in the liver (1.7 cm in diameter), right colon, left iliac lymph-nodes, and prostate (Figure 1).

**Figure 2:**
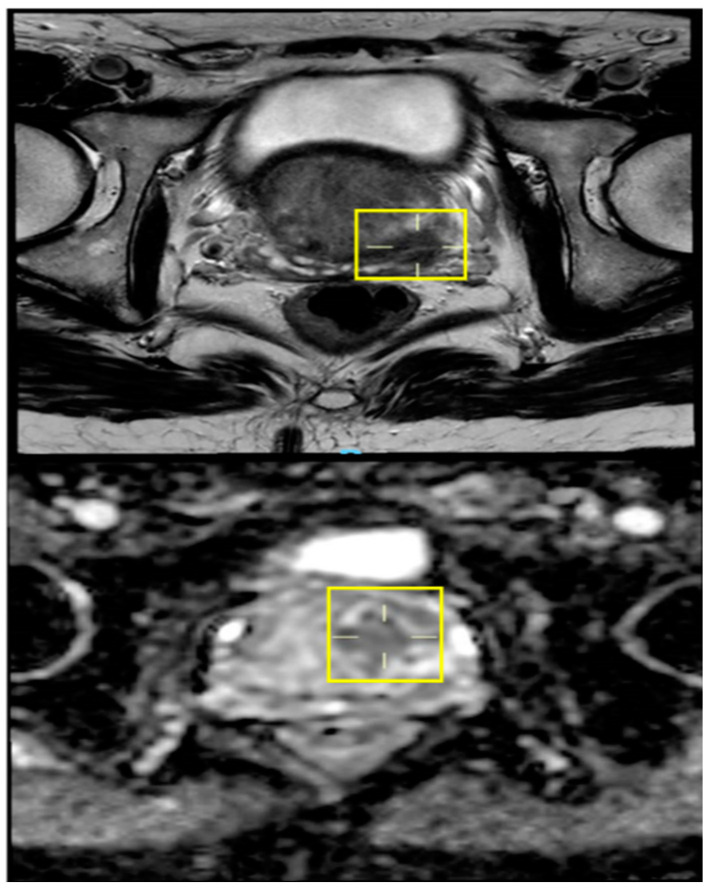
Images from the patient’s MRI with the colon cancer metastasis to the area of the prostate (yellow squares).

A second primary prostate cancer was suspected upon further investigation. As a result, prostate-specific antigen (PSA) was measured and found to be elevated at 6.2 ng/mL (normal 0 to 2.5 ng/mL). Magnetic resonance imaging (MRI) of the pelvis showed a mass over the seminal vesicle, and prostate cancer could therefore not be excluded (Figure 2).

The patient underwent a prostatectomy with histopathological analysis showing adenocarcinoma of colonic origin. The patient is currently undergoing intermittent chemotherapeutic treatment (August 2023).

### 2.2. A Male with Colon Cancer Metastasized to the Prostate Gland

The patient is a 73-year-old male with a 25 PY smoking history ischemic heart disease, diabetes mellitus, chronic kidney disease, hypertension, and hyperlipidemia. He denied any family history of cancer. The patient complained of constipation and abdominal pain, warranting an investigative analysis of the abdominal region. In September 2009, he was diagnosed with stage 3 adenocarcinoma of the sigmoid colon (T3 N1 M0).

**Figure 3:**
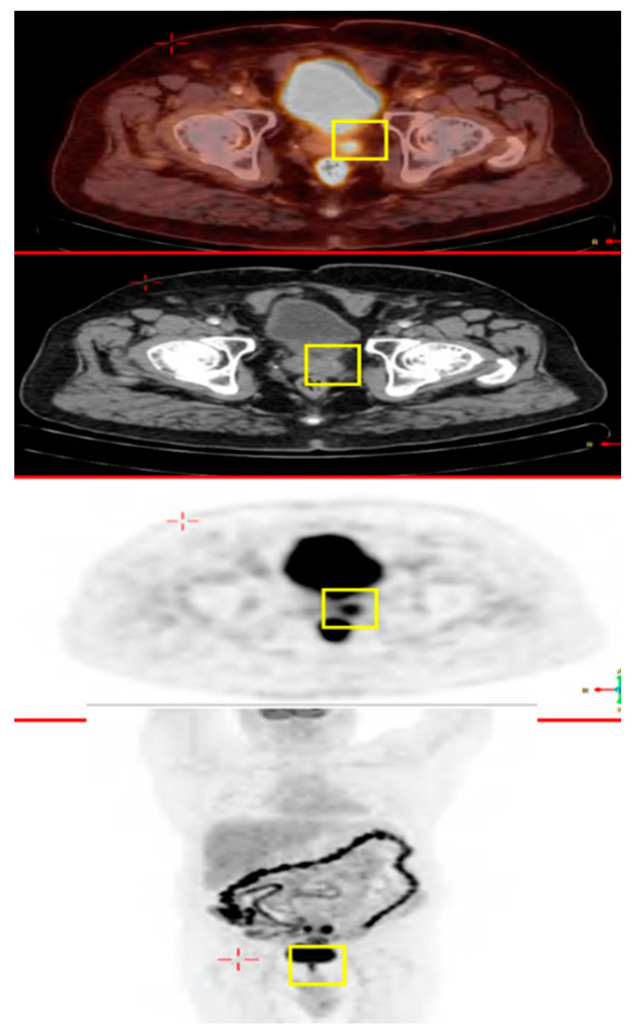
Images from the patient’s PET-CT showing the hyper-metabolic uptake prostate in the area of the (yellow squares).

The patient underwent a sigmoidectomy with negative surgical margins. He was also given a similar chemotherapy regimen based on the FOLFOX protocol (IV oxaliplatin 85 mg/m^2^, leucovorin 400 mg/m^2^ and 5-FU 400 mg/m^2^ on day 1, followed by continuous IV infusion of 5-FU 2400 mg/m^2^ adjuvant therapy over 46 h every two weeks) for six cycles. In December 2014, PET-CT showed hypermetabolic uptake in the area of the prostate (Figure 3). PSA was measured in order to potentially rule out a second primary prostate tumor and was found to be within normal limits (2 ng/mL). The patient subsequently underwent a prostatectomy.

Histopathological analysis revealed adenocarcinoma of colonic origin. The patient is currently undergoing intermittent chemotherapeutic treatment (July 2023).

### 2.3. A Male with Pancreatic Cancer Metastasized to the Right Ventricle of the Heart

The patient is a 63-year-old male with a 35 PY smoking history, dyspnea, and dyslipidemia. He has no family history of cancer. As a result of shortness of breath, a CT angiography of the chest was performed, which showed pulmonary embolisms in both lungs. A CT of the chest and abdomen showed a mass in the head of the pancreas, secondary growth patterns in the liver, and pulmonary emboli in the segmental branches of the pulmonary arteries. A consolidation in the right lower lung appeared to be the result of an infarct.

A liver biopsy performed under ultrasound guidance showed metastatic adenocarcinoma of unknown primary origin. However, consideration of the pancreato-biliary system was warranted. Laboratory results showed elevations in liver enzymes (GGT 426, ALT 46, AST 53, ALP 337) and no increase in bilirubin. Cancer markers were also elevated, namely CA-125 (2072), CA-19-9 (386034), and CEA (471). Due to left chest pain, an echocardiogram was performed, which showed normal left ventricular systolic function, preserved right ventricular function, mild tricuspid regurgitation, and severe pulmonary hypertension.

As part of further investigation into a likely malignancy, PET-CT showed a hypermetabolic region in the head of the pancreas indicative of a primary tumor. Additionally, there was a hypermetabolic mass between the pancreas and the third part of the duodenum. Finally, metastases were identified via increased hypermetabolic activity throughout the liver and portacaval lymph nodes. A distant bone metastasis was likewise discovered in the front part of the fourth rib with sclerotic changes. The apical myocardium was also found to have a possible metastasis from the same scan.

**Figure 4:**
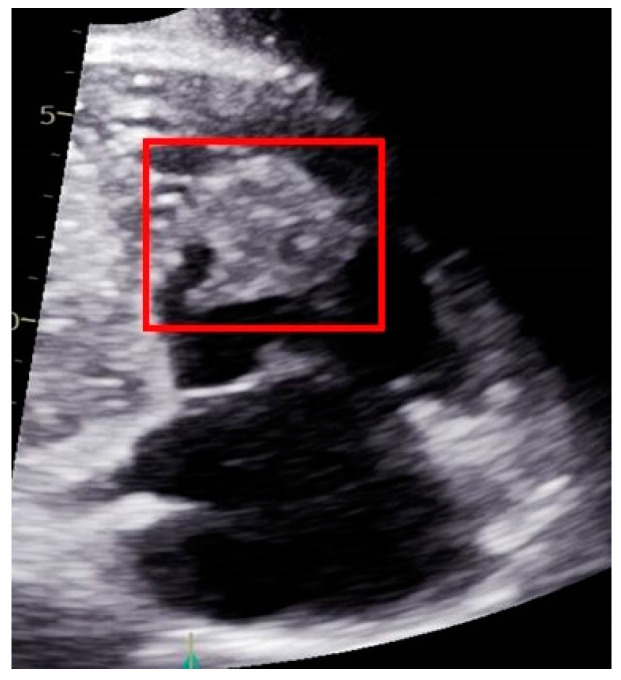
Images from the patient’s echocardiograph showing a cardiac mass in the right ventricle (red square).

Therefore, the patient underwent an echocardiograph, which revealed a mass in the right ventricle of the heart with a diameter of 4.3 cm × 3.5 cm (Figure 4). Based on the scan results, two primary considerations were made: the possibility of a cardiac myxoma or a metastatic tumor. The treatment approach for cardiac myxomas typically involves surgical resection, as it is the preferred method for addressing this condition. However, in this particular case, given the patient’s metastatic disease status and the fact that the myxoma was asymptomatic, the decision was made to initiate chemotherapy instead of opting for surgical intervention.

**Figure 5:**
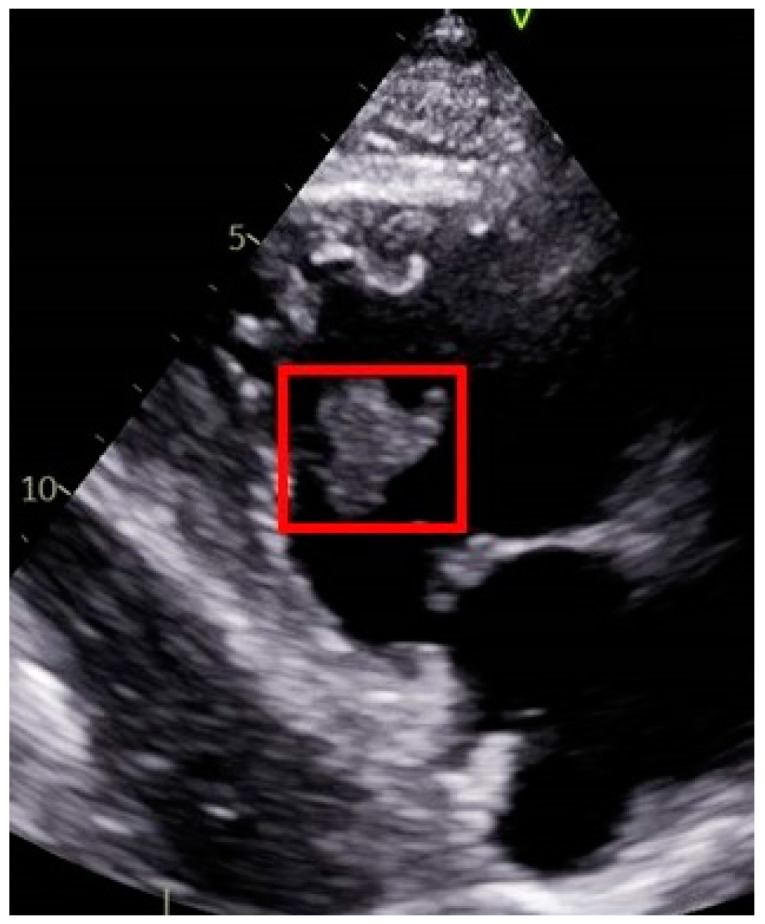
Images from the patient’s echocardiograph showing a decreased diameter of the cardiac mass from the previous scan (red square).

Treatment with the first cycle of the 75% FOLFIRINOX protocol (an IV regimen incorporating irinotecan 250 mg/m^2^, leucovorin 400 mg/m^2^ and 5-FU 400 mg/m^2^ on day 1, followed by a continuous IV infusion of 5-FU 2400 mg/m^2^ over 46 h every two weeks) was ultimately attempted. After two cycles of FOLFIRINOX treatment, echocardiography of the heart was performed for follow-up, which showed a decrease in the diameter of the heart mass to 1.8 cm × 1.6 cm (Figure 5). This outcome gave credence to the hypothesis that the cardiac mass was metastatic in nature.

Following a multidisciplinary consultation involving an oncologist, cardiologist, and radiologist, a consensus was reached that the observed response provided a substantiated indication that the cardiac mass was likely a result of metastasis originating from the pancreas.

### 2.4. A Male with Melanoma Metastasized to the Pancreas

The next patient is a 68-year-old male with a prior history of sliding hiatal hernia, irritable bowel syndrome, benign prostatic hyperplasia (BPH), hypertriglyceridemia, lumbar disc hernia, inguinal hernia, and depressive disorder. For these conditions, he was appropriately being treated with aspirin, omeprazole, atorvastatin, escitalopram, tamsulin hydrochloride, lorazepam and ramipril. The patient does not smoke or drink alcohol.

**Figure 6:**
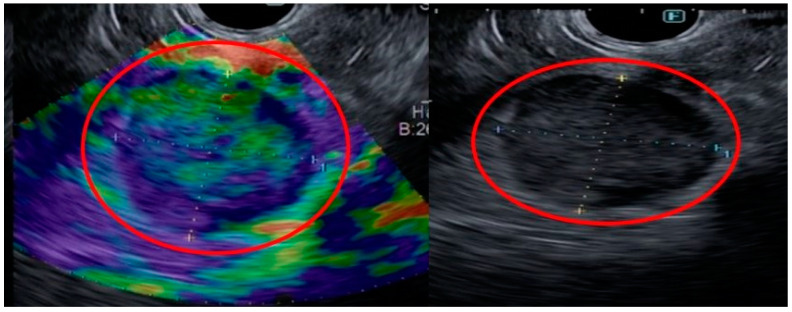
Images from the patient’s endoscopic ultrasound showing a pancreatic mass (red circles) with Doppler (**left**) and without (**right**).

The patient presented with a cutaneous mass in his groin, which was misdiagnosed as an epidermal cyst. He followed up with complaints of a growing mass in his groin, which was later found to be an enlarged lymph node. As a result, further evaluation was warranted. A CT of the abdomen showed enlarged lymph nodes and a possible tumor in the pancreas. Further CT showed nodules in the right lower lung (which were suspected to be a metastasis), a mass in the head of the pancreas (which were was suspected to be a primary tumor), and diffuse retroperitoneal, left groin and pelvic masses up to 2.5 cm in diameter. A biopsy under endoscopic ultrasound was performed on the pancreatic mass, which showed metastatic malignant melanoma (Figure 6).

In addition, an excisional biopsy of the left groin lymph node showed metastatic melanoma. Therefore, the diagnosis of metastatic melanoma with malignant tumors in the lung, pancreas, and lymph nodes was appropriate. Treatment was performed according to ECOG guidelines. Specifically, he was given the first round of immunotherapy (ipilimumab 3 mg/kg plus nivolumab 1 mg/kg), which was well tolerated with minor complaints.

### 2.5. A Male with Breast Cancer Metastasized to the Left Kidney

This 73-year-old male patient has relevant disease history and risk factors. He was a cigarette smoker (20 PY) and had been treated appropriately for hypertension, hypothyroidism, ischemic heart disease and atrial fibrillation. He had a family history of cancer as well, as his mother was diagnosed with colon cancer at age 80, his sister was diagnosed with breast cancer at age 50, and two nephews were diagnosed with lymphoma at ages 8 and 15, respectively.

The patient was also diagnosed with glomerulosclerosis in 2004. He therefore underwent a right kidney transplant in 2012, which failed, and a left kidney transplant in 2013, which succeeded.

**Figure 7:**
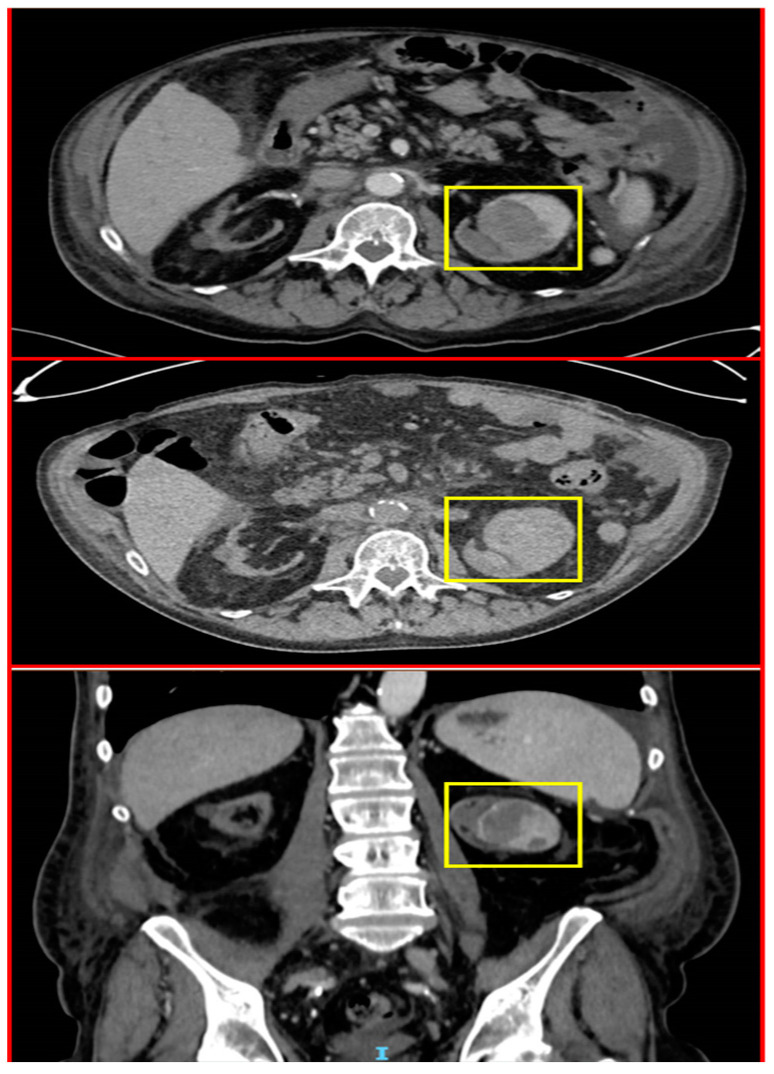
Images from the patient’s CT during investigation of hematuria showing a mass in the upper pole of the left kidney (yellow squares).

In November 2018, he presented with complaints of hematuria and subsequently underwent an abdominal CT, revealing a mass in the upper pole of the left kidney (4.7 cm × 5.4 cm). As part of further investigation, the patient underwent CT urography that confirmed the location of the mass in the upper pole of the left kidney, as well as blood clots in the renal pelvis and proximal urethra, bilateral pleural effusions, and traces of free fluid in the abdomen and pelvis (Figure 7). Renal cell carcinoma was suspected, and the patient underwent nephrectomy of the left kidney.

**Figure 8:**
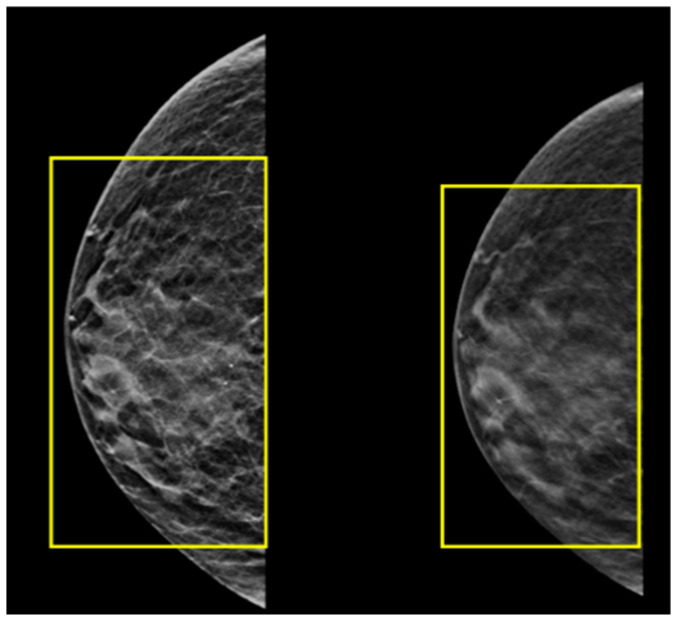
Images from the patient’s ultrasound and mammography screening of the left breast showing prominent breast tissue throughout (yellow squares).

**Figure 9:**
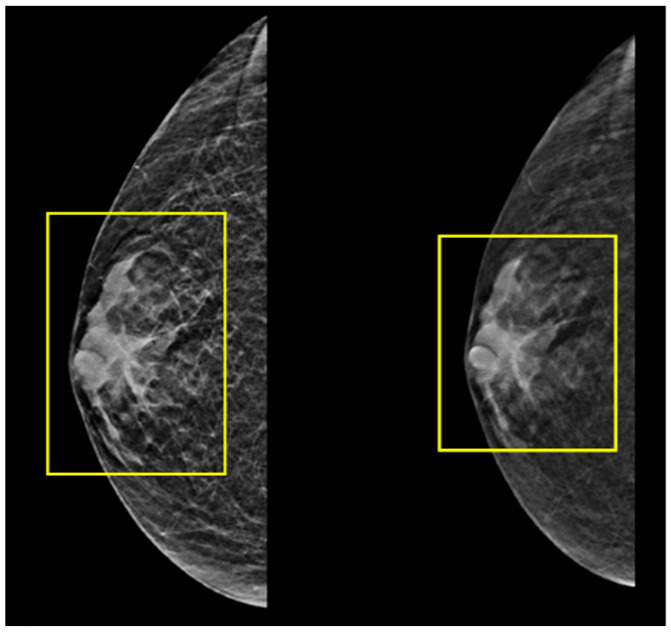
Images from the patient’s ultrasound and mammography screening of the right breast showing prominent breast tissue throughout (yellow squares).

Histopathological analysis of the excised mass was highly suggestive of a breast tissue origin. Analysis of the hormone receptor status of the tumor resulted in a designation of estrogen and progesterone receptor as positive but human epidermal growth factor receptor 2 (HER-2) as negative. Ultrasound and mammography of the left breast showed prominent breast tissue throughout (BIRADS 2) (Figure 8), with similar findings in the right breast (BIRADS 2) (Figure 9), suggestive of benign findings bilaterally. The patient then underwent PET-CT, which did not reveal any areas of hypermetabolic activity. The patient is currently being treated with tamoxifen (Nolvadex). His last PET-CT (July 2023) showed no evidence of further metastatic disease. Due to the rarity of this case and the fact that there is a history of organ transplantation in this patient, a relevant analysis was conducted on the medical background of the kidney donor. It is particularly noteworthy, therefore, that this investigation revealed that the donor did not have any personal or family history of cancer. In addition, no mutations or biomarkers were found.

## 3. Discussion

Given the importance of statistics in medicine, case studies of rare metastatic cancer have value for scientists and medical professionals. We presented rare oncological metastases from five patients (Table 1).

The first two patients were diagnosed with colonic adenocarcinoma, with follow-up PET-CT demonstrating areas of hypermetabolic activity in the prostate. These prostate tumors were presumed to be secondary cancers. However, histopathologic analysis found them to be metastatic tumors of colonic origin. One of the patients initially refused prostatectomy and instead requested treatment according to the prostate cancer protocol. However, after consideration, he agreed to surgical intervention. Of note were the inconsistent PSA findings between these patients. Namely, neither had continuing metastatic pancreatic disease, although one patient (patient 1) had an elevated PSA of more than twice the upper limit of normal, while the other patient (patient 2) had levels within the normal range. One possible explanation is that PSA levels tend to rise with factors such as age, infection, trauma, and inflammation. Additionally, benign prostatic hyperplasia (BPH) can lead to increased serum PSA levels, which can reduce the reliability of this biomarker for predicting prostate cancer. Studies have indicated that as many as 86% of individuals with BPH may exhibit elevated serum PSA levels. Interestingly, research has revealed that PSA is expressed not only in prostatic tissues but also in various non-prostatic tissues. As a result, there is growing interest in exploring its potential as a biomarker for cancers beyond those of the prostate. Notably, elevated PSA levels have been associated with colorectal and breast cancer, further expanding its potential applications in cancer diagnosis [43,44,45]. The next two patients were diagnosed with pancreatic adenocarcinoma with metastases in the heart, bone, liver, and lymph nodes and metastatic melanoma with metastases in the pancreas, lung, brain, and lymph nodes, respectively. As is often the case with pancreatic cancer, the patient with pancreatic adenocarcinoma only presented with symptoms as a result of distant metastasis, specifically dyspnea in this case. The patient with metastatic melanoma had a skin lesion that was initially misdiagnosed. This was not resolved until he presented with an additional enlarging mass in the neck, later found to be a lymph node metastasis of melanoma. Both patients are being treated with the appropriate medical interventions as specified by ECOG guidelines. The final patient was diagnosed with metastasis from male breast cancer that was initially suspected to be renal cell carcinoma. Histopathology of the renal mass confirmed a metastasis of breast tissue origin. The patient subsequently underwent a treatment protocol in accordance with the carcinoma’s hormonal status.

Pancreatic adenocarcinoma is often considered a metastatic disease, as most cases present with local and metastatic recurrences and only 10–15% of cases are surgically resectable [34,46]. While the most common sites are the liver (80%), peritoneum (48%), and lungs (45%), metastases may often also be found in the bones (5%), and distant lymph nodes (9%) [47]. Cardiac metastases are very rare and generally found postmortem [48]. In our patient’s case, follow-up echocardiography after 2 cycles of treatment revealed a decreasing diameter of the mass from 4.3 cm × 3.5 cm to 1.8 cm × 1.6 cm. This outcome strengthens the hypothesis that the mass was a metastasis from the pancreatic adenocarcinoma, as myxomas do not respond to chemotherapy while metastatic disease often can. This is why the only way to treat myxomas is removal through surgical resection. Furthermore, there is a hypothesis suggesting that cardiac trauma and the subsequent local inflammation might play a role in the development of myxomas, which is not relevant for our patient [49]. Due to the status of metastatic disease in this patient, it was preferred to start chemotherapy instead of surgical interventions. The initial response was a positive indicator for further treatment. Metastatic melanoma presents a wide-ranging metastatic profile. Metastatic disease is frequently observed in the lymph nodes (73.6%), lungs (71.3%), liver (58.3%). Brain (49.1%), bone (48.6%), heart (47.2%), adrenal glands (46.8%), and gastrointestinal (GI) tract (43.5%) [50]. Malignant metastatic melanoma accounts for only 2% of all pancreatic cancers [51]. Indeed, it is typical for renal, breast, lung, and colon cancers to metastasize to the pancreas, while occurring far less commonly in malignant melanoma [51].

Although breast cancer is known as a rare disease in male patients, the authors suggest that its metastases should be considered in various peripheral sites, including regions previously unsuspected and those recognized to have rare occurrences [36,37,38,39,40,41]. In addition, it is important to further consider the possibility of rare metastases when assessing patients with prostate concerns with a suspicion of malignancy.

As demonstrated, a comprehensive investigation should be conducted when there are unusual findings to rule out any rare occurrences. Our review of the current literature yielded only five reported cases of patients with colon cancer with confirmed metastases to the prostate gland. The first was diagnosed in 1993, and the last was reported in 2013 (Table 2) [9,10,11,12,13].

The “seed and soil hypothesis” underpins the understanding of metastatic dissemination to specific organs. In this conceptual framework, tumor cells, likened to “seeds”, release cytokines and proteins that interact with the microenvironment of distant sites referred to as the “soil”. Effective interaction between these cytokines and the microenvironment facilitates the homing of circulating tumor cells to distant locations, thus inducing metastasis. However, certain sites, such as the skin, spleen, and muscles, are less conducive to distant metastasis. Factors contributing to this may encompass reduced cardiac output, particularly for the skin; the presence of immune cells and immune surveillance in organs like the spleen; or an unsupportive environment due to factors such as low pH or anaerobic metabolism, as is characteristic of muscles.

The atypical occurrence of metastasis at unusual sites can complicate the differentiation between a metastatic lesion and a second primary tumor. Radiologists and pathologists play a pivotal role in resolving this diagnostic ambiguity. Determining the distribution pattern of metastatic lesions, recognizing morphological similarities between sites of metastases, and, in more complex cases, conducting arterial and venous phase studies contribute to establishing the diagnosis of metastasis at unconventional locations [52]. In instances where the metastasis is solitary or where the nature of the lesion is challenging to ascertain, an additional tissue biopsy from the uncommon site may be necessary to confirm the presence of metastatic disease.

As a general observation, encountering solitary rare metastases is infrequent; the overwhelming majority of rare metastatic occurrences tend to coincide with the emergence of metastases in other locations, underlying a progression towards more extensive systemic disease.

In the realm of research, recent strides in comprehending the cellular and molecular intricacies of the metastatic process offer unparalleled potential for enhancing and formulating effective supplementary treatment approaches.

On the clinical front, timely identification of secondary lesions remains the sole opportunity to manage the disease and extend survival. Consequently, familiarity with both common and uncommon sites of metastases empowers physicians to discern symptoms promptly and strategize optimal treatment plans.

Of paramount importance is to consider that a better understanding of the metastatic process may reveal more therapeutic targets for future oncological treatment.

## 4. Conclusions

Our reported article is the first to describe a male with metastatic breast cancer in the kidney. Moreover, we present two unique occurrences of metastases to the prostate that contribute to the growing body of literature describing less common metastatic findings. Finally, we describe rare metastases from pancreatic adenocarcinoma located in the heart and metastatic melanoma spreading to the pancreas. The follow-up of these patients can yield valuable information for the future treatment of other such cases with long odds of success.

## Figures and Tables

**Table 1:** Patient summary table of rare metastatic diseases.

Patient No.	Age at Diagnosis	Gender	Diagnosis	Site of Rare Metastasis	Diagnostic Method	Treatment *
1	69	M	Colorectal Adenocarcinoma	Prostate	Histopathology	FOLFOX
2	73	M	Colorectal Adenocarcinoma	Prostate	Histopathology	FOLFOX
3	63	M	Pancreatic Adenocarcinoma	Heart	PET-CT Histopathology	FOLFIRINOX
4	68	M	Metastatic Melanoma	Pancreas	CT Histopathology	Ipilimumab/Nivolumab
5	73	M	Breast Carcinoma	Left Kidney	CT Histopathology	Resection Tamoxifen

* While this was ultimately the treatment plan decided, it should be clear that a greater cohort must be studied in order to determine the best possible outcomes and that variations may be warranted based on other health variables.

**Table 2:** The basics of the previous reported cases for male patients with colon cancer that had metastasized to the prostate.

Date of Publication	Age (Years)	Stage at Presentation	Reference No.
February 1993	N/A *	N/A	[17]
August 2002	N/A	N/A	[18]
May 2004	38	IIIC	[19]
June 2011	54	IIIC	[20]
June 2013	70	IV	[21]

* N/A indicates to information was available.

## Data Availability

Data are contained within the article or are available from the authors upon reasonable request.
